# A hybrid intelligent method for three-dimensional short-term prediction of dissolved oxygen content in aquaculture

**DOI:** 10.1371/journal.pone.0192456

**Published:** 2018-02-21

**Authors:** Yingyi Chen, Huihui Yu, Yanjun Cheng, Qianqian Cheng, Daoliang Li

**Affiliations:** 1 College of Information and Electrical Engineering, China Agricultural University, Beijing, China; 2 Key Laboratory of Agricultural Information Acquisition Technology, Ministry of Agriculture, Beijing, P.R. China; 3 Beijing Engineering and Technology Research Center for Internet of Things in Agriculture, Beijing, P.R. China; Tokai University, JAPAN

## Abstract

A precise predictive model is important for obtaining a clear understanding of the changes in dissolved oxygen content in crab ponds. Highly accurate interval forecasting of dissolved oxygen content is fundamental to reduce risk, and three-dimensional prediction can provide more accurate results and overall guidance. In this study, a hybrid three-dimensional (3D) dissolved oxygen content prediction model based on a radial basis function (RBF) neural network, K-means and subtractive clustering was developed and named the subtractive clustering (SC)-K-means-RBF model. In this modeling process, K-means and subtractive clustering methods were employed to enhance the hyperparameters required in the RBF neural network model. The comparison of the predicted results of different traditional models validated the effectiveness and accuracy of the proposed hybrid SC-K-means-RBF model for three-dimensional prediction of dissolved oxygen content. Consequently, the proposed model can effectively display the three-dimensional distribution of dissolved oxygen content and serve as a guide for feeding and future studies.

## Introduction

Dissolved oxygen content plays a vital role in aquatic ecosystems because it has a substantial influence on water quality management, feed consumption, and energy expenditure [1]. Proper control and management of dissolved oxygen content in crab pond aquaculture is crucial for the developing crabs and has a significant impact on the quality and quantity of the final product [2]. An inappropriate dissolved oxygen content can cause crab hypoxia and even more severe disease [3]. Therefore, establishing an efficient and accurate model for predicting dissolved oxygen content in crab aquaculture is important to provide a basis for water quality control and reduce aquaculture risk. Liu et al. and Yu et al. built dissolved oxygen content prediction models based on the machine learning method, which achieves time dimension forecasting without considering three-dimensional prediction [2, 3]. However, a three-dimensional prediction model for dissolved oxygen content can reveal changing trends and provide guidance for aquaculture. Therefore, in this study, we propose a hybrid three-dimensional (3D) dissolved oxygen content prediction model that can achieve more accurate results and overall guidance for dissolved oxygen content in crab ponds.

Many artificial methods have been applied to water quality prediction and achieved significant results, including the water quality index [4], artificial neural networks [5], and support vector regression methods [6]. Water quality assessment models based on dynamic analysis methods have been applied to lakes [7] and rivers [8] to precisely assess impact factors for water quality. Although the dynamic factor mode enables long-term water quality analysis and prediction, it is not suitable for the nonlinear problem and cannot provide precise prediction accuracy [9]. Artificial neural networks and support vector regression methods not only solve nonlinear prediction but also improve prediction accuracy [10]. A back propagation neural network (BP-NN) has been used to predict the water quality in intensive aquaculture water management [11]. Dissolved oxygen content is one of the most important parameters in the characterization of the water condition [12]. Artificial neural networks have been successfully applied in a number of studies focused on dissolved oxygen content prediction. Anita et al. applied three alternative neural network models radial basis function neural network (RBFNN) and multivariate linear prediction neural network (MLPNN) and a multivariate linear regression model (MLR) for dissolved oxygen content prediction in river water and showed that the best performing models were GRNN and RBFNN [12]. A RBFNN model was developed to predict the dissolved oxygen from biochemical oxygen demand (BOD) and chemical oxygen demand (COD) in the Surma River [13]. Samira and Salim developed an adaptive neural based fuzzy inference system [14] and dynamic evolving neural-fuzzy inference system [15] for modeling river dissolved oxygen concentration. Recently, a new extreme learning machine model was applied to predict dissolved oxygen concentration with and without water quality variables as predictors. The ability of ELM to outperform MLPNN and MLR for modeling DO with and without water quality variables as predictors was assessed [16]. Additionally, Liu et al. proposed a dissolved oxygen prediction model based on the least square support vector regression (LSSVR) optimized by particle swarm organization [3]. Various computational intelligence techniques (e.g., MLP, RBF, LGP, and SVM) for estimating DO concentration were compared by Ehsan et al. The SVM provided the most accurate model for DO estimation in comparison to other models [17]. An intelligent controller based on an evolving fuzzy neural network was applied to the automatic and intelligent detection of breakpoints in dissolved oxygen (DO) profiles [18]. However, the above studies are limited to one-dimensional prediction for dissolved oxygen content, which cannot provide detailed guidance for dissolved oxygen content in crab ponds. Compared to one-dimensional models, three-dimensional models are a more powerful means of visualizing, analyzing and accurately predicting dissolved oxygen content in an aquaculture pond. Hence, this study proposes a three-dimensional model for dissolved oxygen content.

Recent research on the construction of three-dimensional models has primarily focused the inverse distance weighting and Kriging methods of spatial interpolation [19, 20]. However, these methods have poor adaptive variation and require numerous assumptions, and the spatial interpolation accuracy depends on the number of samples [21]. Consequently, RBFNNs have been used in spatial interpolation problems due to its strong nonlinear fitting ability. RBF networks, which can be viewed as a computer model, imitate the learning capabilities of the human brain [22]. The Kriging interpolation method has become a favored interpolation routine and has been used by many environmental scientists for interpolation and mapping [20]. Artificial neural networks have demonstrated higher accuracy than Kriging for spatial mapping of complex patterns of arsenic contamination of groundwater because neural networks are more suitable for nonlinear problems [23]. In this study, the time dimension was added to the dissolved oxygen content prediction because aquaculture water is an open, nonlinear, dynamic and complex system [3]. Therefore, RBF was selected as a three-dimensional model for dissolved oxygen prediction. RBF-based spatial interpolation involves the optimal estimation of parameters: numbers of hidden nodes, the hidden layer’s center value and width, and the link weight. The number of hidden nodes and center value have typically been considered difficult to determine in radial basis function neural networks [24]. To improve the accuracy, Liu Sicong adapted the integrated RBF neural network for spatial data interpolation of lead content [25]. I-Cheng Yeh et al. concluded that both MLP and RBF have advantages and disadvantages in spatial interpolation, and neither MLP nor RBF can easily construct a model with sufficient accuracy to fit a plane-peak hybrid surface. Thus, they combined MLP and RBF and proposed a hybrid neural network with both sigmoid and Gaussian functions as the hidden layer. However, these previous studies have neglected the question of how to determine the hidden layer nodes and center value in the RBF spatial interpolation method.

In this study, SC-K-means-RBF 3D spatial interpolation of dissolved oxygen content in aquaculture ponds is presented. The fundamental process of the SC-K-means-RBF 3D spatial interpolation is as follows: first, the initial cluster center *C*_*S*_ and cluster number *N*_*S*_ of the sampling data are calculated with subtractive clustering; second, based on the initial cluster center *C*_*S*_ and cluster number *N*_*S*_, the ideal cluster center *C* is obtained by K-means clustering; and finally, the dissolved oxygen content values of all points in the entire aquaculture pond can be interpolated by using the obtained parameters to design the radial basis neural network and then a 3D model of the dissolved oxygen content of the aquaculture pond.

The remainder of this paper is structured as follows. Section 2 describes the basic methodology. Section 3 presents the parameter selection of RBF with the SC-K-means method and the overall description of the three-dimensional prediction of dissolved oxygen content. Section 4 provides the prediction results of the hybrid method and the three-dimensional model performance. Section 5 gives the conclusions from this research and suggests directions for future research.

## Methodology

### Standard RBF neural network

**Fig 1:**
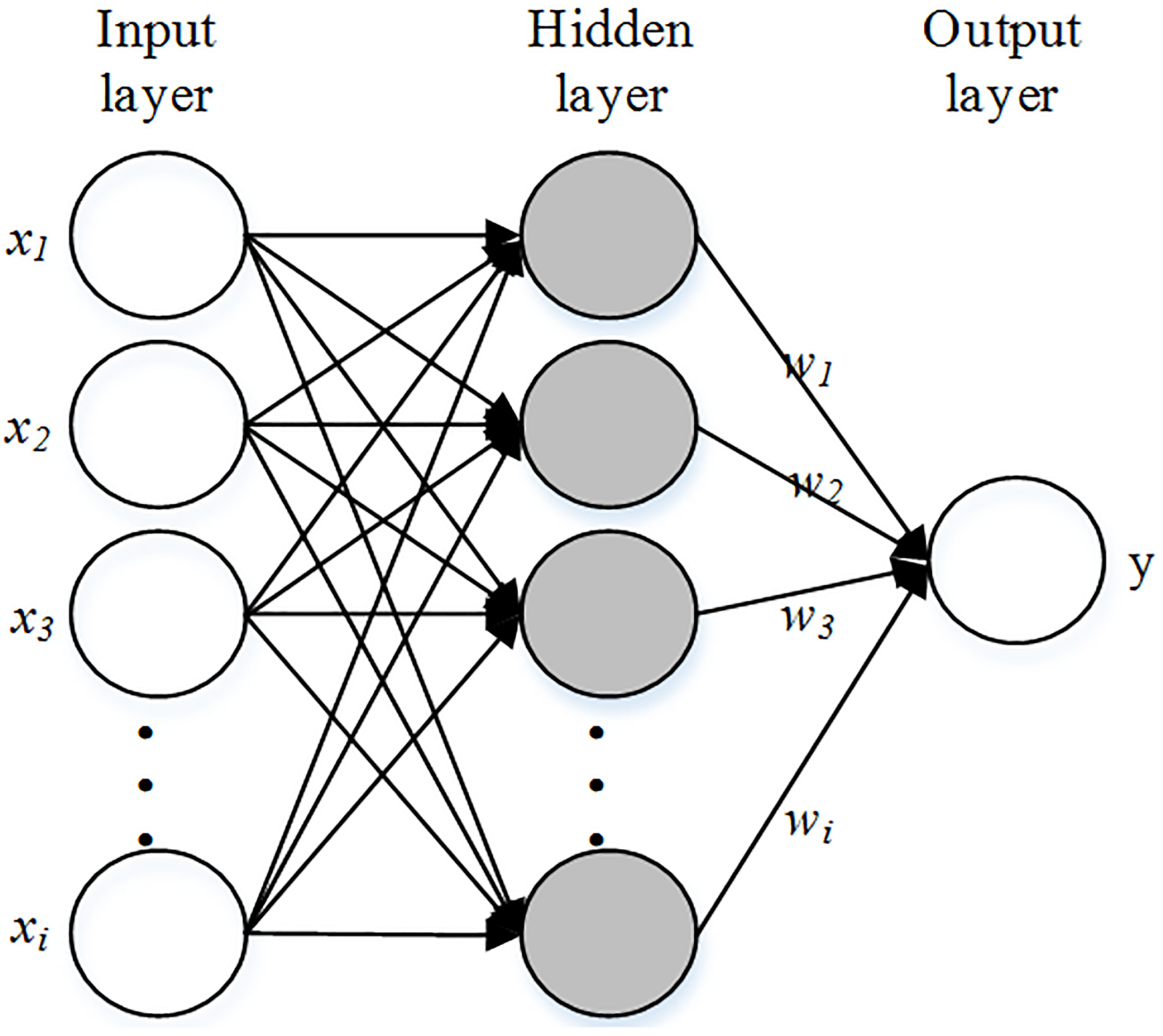
Structure of RBF[22].

The RBF neural network, which was presented by C. Darken and J. Moody in 1989, is one of the most widely used neural network models [26]. This neural network has an uncomplicated structure with three layers: the input layer, the hidden layer, and the output layer, as shown in Fig 1. The input data X are an m-dimensional vector, where X = [*x*_1_, *x*_2_ … *x*_*m*_]^*T*^. The input data are transmitted to the hidden unit by the input unit [27]. The hidden unit response for the input data consists of local activation functions [25]. The response has the following form:

$$\varphi_{i}(x_{j})\;=\;\varphi (\left\|x_{j}-c_{i}\right\|)\;i\;=\;1,2,\ldots ,n$$ (1)

where *φ*_*i*_ denotes the *i*th hidden unit’s response, *x*_*j*_ is the *j*th input data, *c*_*i*_ denotes the center of the *i*th unit, *φ*(*x*) denotes the activation function, *n* denotes the hidden node’s number, and ||x_j_−c_i_|| denotes the Euclidean norm. There are different types of functions *φ*(*x*) that satisfy the definition, and the Gaussian function has the following form:

$$\varphi (X)\;=\;\text{exp}(-\frac{{\left\|X-c_{j}\right\|}^{2}}{2\sigma^{2}})$$ (2)

where σ denotes the hidden layer’s width, which can be calculated by

$$\sigma \;=\;\frac{d_{max}}{\sqrt{2n}}$$ (3)

where *d*_*max*_ denotes the maximum distance between hidden nodes, and *n* denotes the number of hidden nodes.

For the output layer, there is a linear relationship between the hidden layer and output layer, which can be defined as follows:

$$y\;=\;\sum_{i\;=\;1}^{n}w_{i}\varphi_{i}(X)i\;=\;1,2,\ldots ,n$$ (4)

where *w*_*i*_ denotes the connecting weight value of the *i*th hidden units and the output unit. The training of RBF neural networks is a process of estimating the above parameters, namely the number of hidden nodes *n*, the center value of the hidden nodes *c*, the width σ, and the link weight *w*_*i*_ [27]. Commonly, two-staged learning strategies are used to determine the parameters. In the first stage, the widths and the correct values of the centers are calculated by using the K-means clustering method, and in the second stage, the least squares method is used to calculate the connection weights, which is a standard process [28].

### K-means clustering

Clustering is a process in which sampling data can be divided into many different groups so that each group has some common characteristics [29]. Clustering methods include the K-means clustering algorithm, the multiple kernel K-means clustering algorithm, hierarchical algorithms, and the combined cluster and discriminant analysis method. The K-means algorithm is a practical and classic method of clustering [30]. Hierarchical algorithms are usually used as a baseline or to optimize the base clustering algorithm; these algorithms are computationally intensive and cannot be used for large-scale calculations [31]. The multiple kernel K-means clustering algorithm is an optimized method of the basic K-means algorithm. The combined cluster and discriminant analysis (CCDA) method can search for homogeneous groups and show exactly whether further separation is needed with the difference values attributed to each grouping [32]. Compared to the above methods, the K-means clustering algorithm is simple and effective. Hence, the K-means algorithm was chosen to calculate the hidden nodes of the RBF.

The aim of K-means is to obtain K cluster centers in the sampling data to minimize the sum of the square distance between every datum point *X*_*i*_ and its nearest center [33]. A randomly selected initial K-center and the value of K are required for the K-means clustering algorithm. Let X = {*x*_*i*_|*i* = 1, …, *n*} be a sampling data set, C = {*c*_*i*_|*i* = 1, …, *k*} be a set of K centers selected from X, and *M*_*k*_ = {*m*_*i*_|*i* = 1, … *m*} be a set of samples that belong to the K^*th*^ cluster[34].

The sampling data are assigned to one of the K clusters based on the minimum value of the distance *d*(*x*_*i*_, *c*_*i*_) between the clusters and the sampling datum point. The distance *d*(*x*_*i*_, *c*_*i*_) is given by the following:

$$d(x_{i},c_{j})\;=\;\|x_{i}-c_{j}\|$$ (5)

where ||.|| denotes the Euclidean distance between a sampling data *x*_*i*_ and its cluster center *c*_*j*_. Then, the new cluster centers are calculated according to the following:

$$c_{j}\;=\;\frac{\sum_{x_{i}\in M_{j}}x_{i}}{m}$$ (6)

where *m* denotes the number of data items belonging to the *j*^th^ cluster. This process is repeated until there is no change in the cluster centers. Obviously, a fundamental disadvantage of K-means is that the result of clustering depends on the choice of the initial cluster centers. If the number of cluster centers is over-forecast, fictitious classes will be generated. If a low number of cluster centers is chosen, the quality of the resulting clustering will decrease for different merged classes [31]. Thus, the subtractive clustering method was selected to optimize the K-means algorithm in this study.

### Subtractive clustering

Subtractive clustering was developed to overcome the limitations of the momentum method [35]. The basic idea of subtractive clustering is to determine the center of the cluster according to the data density of each sampling point. Each of the sampling points is assumed to be a potential cluster center, and for input vectors (*X*_1_, *X*_2_, ⋯, *X*_*m*_) belonging to the n dimension, the data density of each vector has the following form[36]:

$$D_{i}\;=\;\sum_{j\;=\;1}^{m}exp[-\frac{{\left\|X_{i}-X_{j}\right\|}^{2}}{{\left(\frac{\alpha }{2}\right)}^{2}}]$$ (7)

where *D*_*i*_ denotes the density, *m* denotes the amount of sampling data, *α* denotes the adjacent field next to this point and is a positive constant, and ||.|| denotes the Euclidean distance. Commonly, the value of *D*_*i*_ will be higher if a datum point *X*_*i*_ has more data around it than other datum points. The datum point $X_{1}^{*}$ that has the highest value of $D_{1}^{*}$ is chosen as the first cluster center after the density of each point is calculated. The first cluster’s effect should be subtracted to calculate the new density values of the second cluster center as follows:

$$D_{i}\;=\;D_{i}-D_{1}^{*}exp(-\frac{{\left\|X_{i}-X_{1}^{*}\right\|}^{2}}{{\left(\frac{\beta }{2}\right)}^{2}})$$ (8)

β=μ*α (9)

To avoiding closely clustered centers, β often equals 1.5 * α. Therefore, when $X_{1}^{*}$ is closer to the first cluster center of the points, its density value is lower. After revising the density of each datum point, the second cluster center can be determined, and the density of each datum point can be revised again. The above process is repeated until a condition δ is satisfied, which is defined as follows:

$$\frac{D_{k}^{*}}{D_{1}^{*}}<\delta$$ (10)

where δ is a significance factor for the results of clustering; if the value of δ is too small, there will be many cluster centers, and if the value of δ is too large, the cluster centers will be too numerous.

### Software

All algorithms used in this study for dissolved oxygen content 3D prediction were programmed in MATLAB 9.0. The method package included the Kriging tool box. The experiment was conducted on a 2.50 GHz Core 5 CPU personal computer with 4.0G memory using Microsoft Windows Sever 2007 edition.

### Parameter selection for RBF with the SC-K-means method

In the RBF interpolation problem, four parameters of the RBF neural network must be determined. Researchers have proposed many training methods to calculate the proper parameters of the RBF neural network. However, most available methods do not provide any rational means of calculating the hidden nodes *n*. The number of hidden nodes determines the accuracy and generalizability of the RBF interpolation[37]. When the RBF network has a large number of hidden units, it can attain zero errors. However, when the applications have excessive sampling data, the size of the network will become impractically large and have poor generalizability. By contrast, when the RBF neural network is small, the errors will increase despite better generalizability. Therefore, the calculation of *n* is an important factor for the design of RBF interpolation. The trial-and-error method is commonly used to determine the proper number of hidden units, but it cannot guarantee the best results and may also be a waste of time.

**Fig 2:**
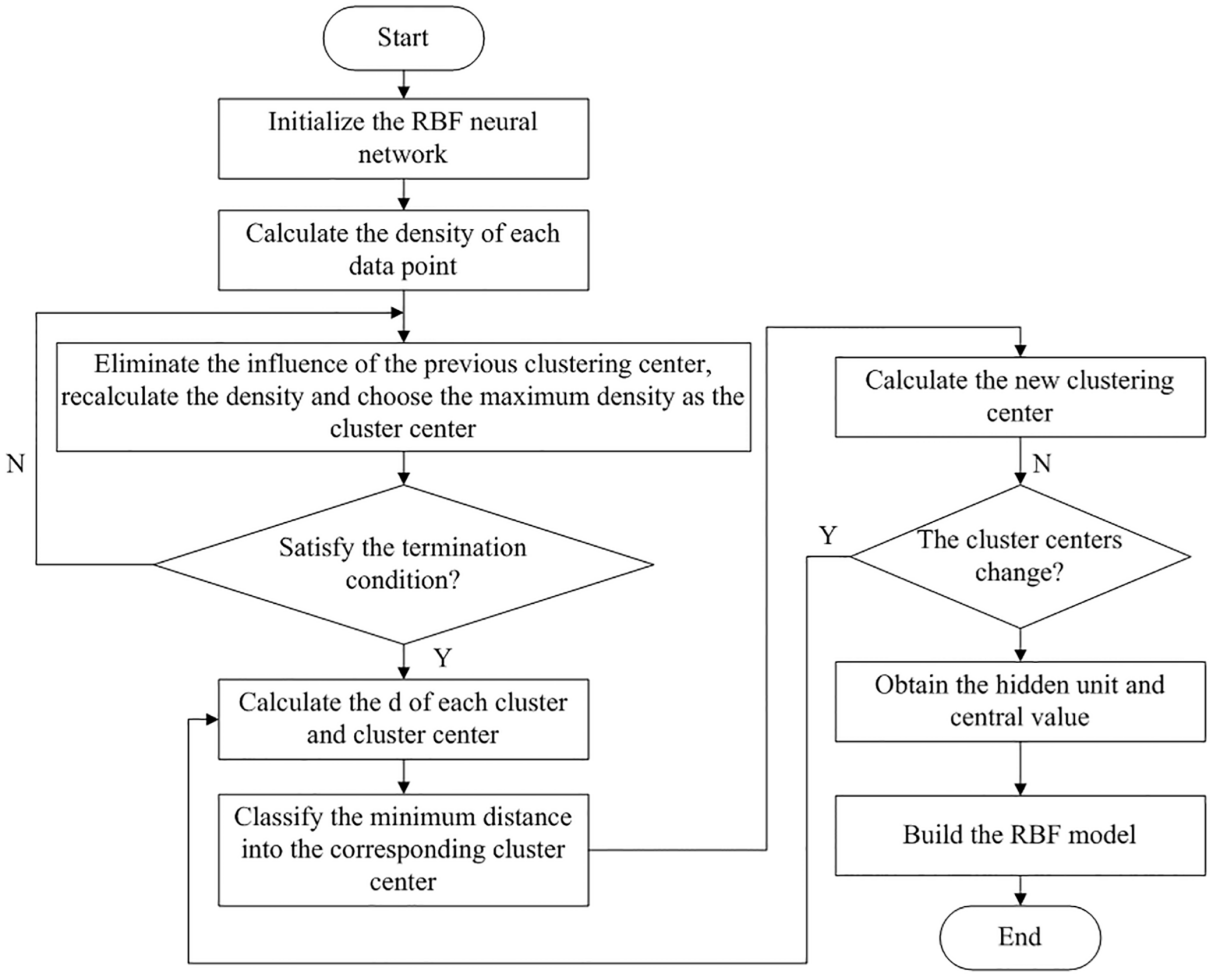
The process of parameter selection for the RBF neural network.

In the radial basis function neural network, the numbers of hidden nodes and center value are normally considered difficult tasks to design. To achieve the best result for selecting the proper number of hidden nodes for the RBF interpolation model, an integrated RBF interpolation method that combines subtractive clustering, K-means, and RBF is proposed and named SC-K-means-RBF. The detailed explanation of the process is as follows (Fig 2):

Step 1. Initialize the RBF neural networks, and then calculate the density of each datum point.
Step 2. The initial cluster center *C*_*S*_ and cluster number *N*_*S*_ of the sampling data can be calculated with the subtractive clustering method.
Step 3. Based on the initial cluster center *C*_*S*_ and the number of clusters *N*_*S*_, the ideal cluster center *C* can be obtained by K-means clustering (e.g., let the parameter K of K-means clustering equal *N*_*S*_ and *C*_*S*_ be the initial cluster center *C*_*c*_ of the K-means cluster).
Step 4. Then, the value of σ can be obtained from the value of *w*_*i*_ using least squares.
  $$\sigma \;=\;\frac{d_{max}}{\sqrt{2n}}$$ (11)
Step 5. Let *n* equal K, and *c* equal *C*_*c*_; then, the four parameters of RBF can be obtained.

## Experimental results and analysis

### Data acquisition and preprocessing

**Fig 3:**
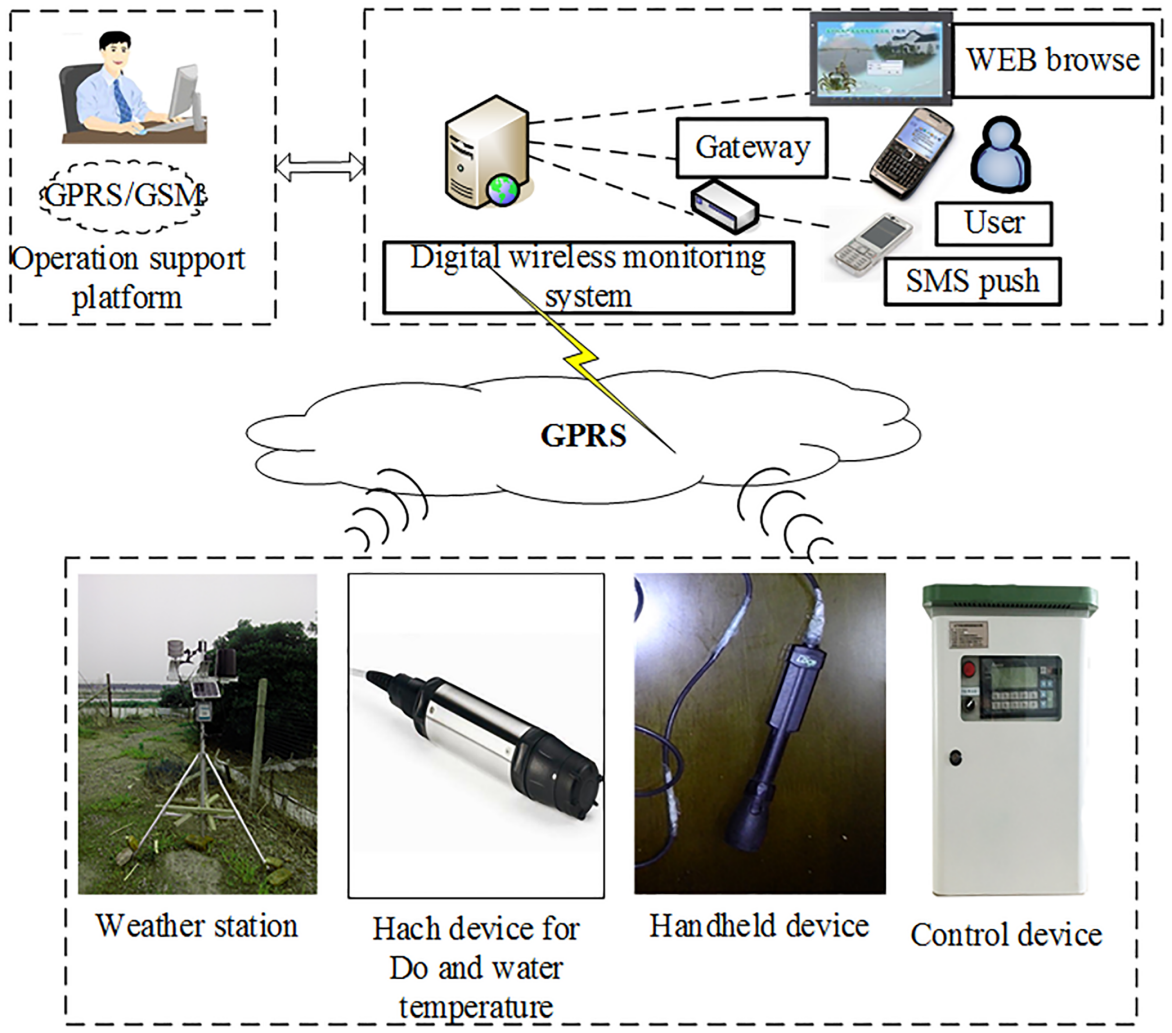
Topology structure diagram of the digital wireless system.

The data used in this study were collected from an aquaculture pond located in Gao Cheng (119.79° *E*, 31.48° *N*), Jiangsu Province, China. The pond is located in the south of Gehulake and has a relatively good spatial environment. The aquaculture pond is 45 meters wide and 130 meters long. The pond is rectangular. The aquaculture pond is open-air. In this area, ponds are close together in a row. In this study, we collected data in two ways: a digital wireless monitoring system and a handheld device. The digital wireless monitoring system comprises three major parts: the water sensors and weather sensors, the transport devices and the application terminal devices (Fig 3). All gathered sensor data are transported to the application layer for data acquisition, intelligent information processing, and logical operation by the transport device.

**Fig 4:**
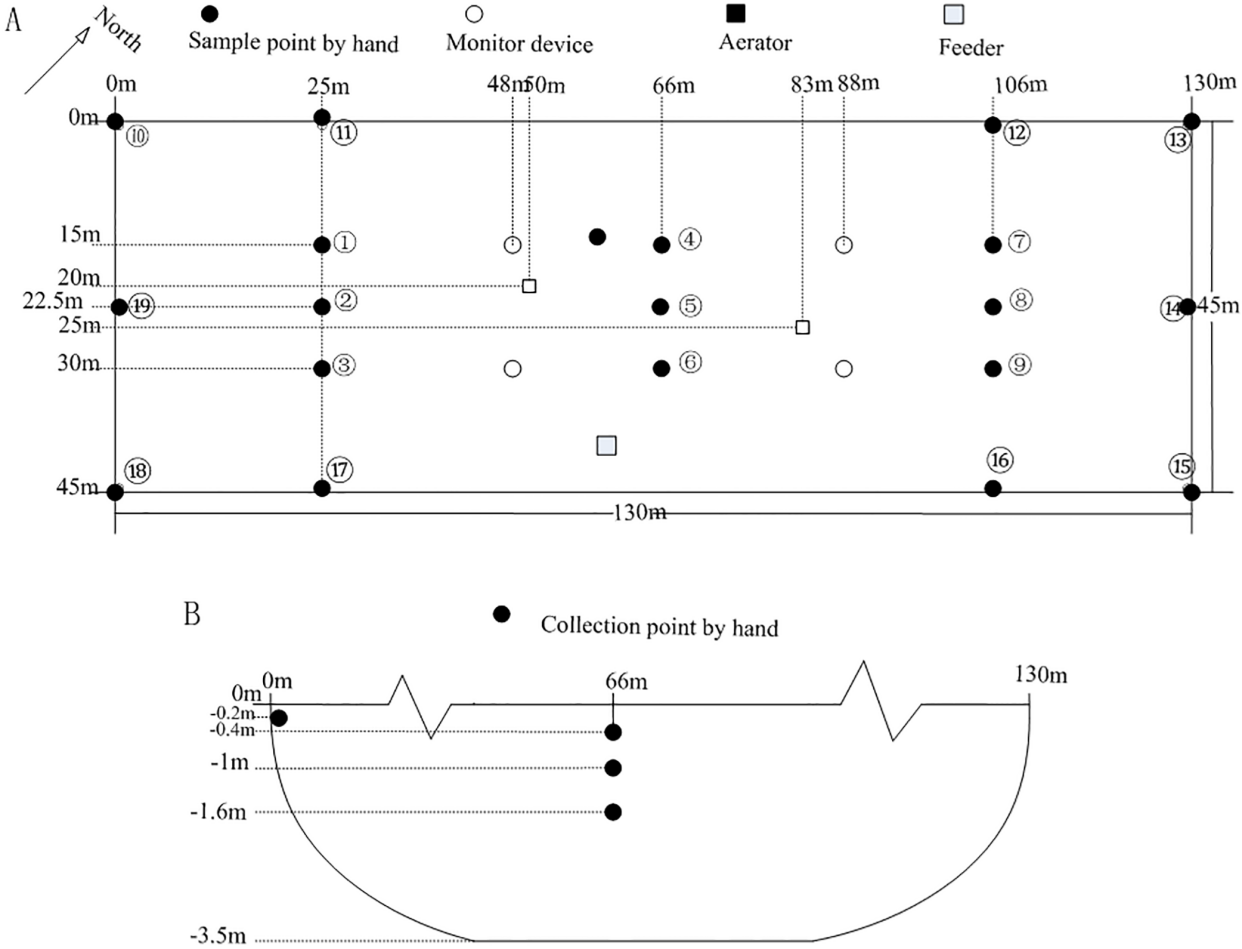
Point distribution of collected samples. Top view of the aquaculture pond (A); sectional view of the aquaculture pond (B).

The details of the device types and accuracy are shown in Table 1. Forty-one positions regularly distributed in the aquaculture pond were selected as the sampling points. The black points represent the aquaculture dissolved oxygen content data collected using the handheld device. The white points represent the aquaculture dissolved oxygen content as measured by the digital wireless monitoring system (Fig 4A).

**Table 1:** Details of the test devices.

Device name	Type	Range	Accuracy
Rainfall collector	Remote transmission weather station Uni-ws-G9 (China)	0.0~9999.0 (mm)	±4%
Wind speed and direction collector		0~67 (m/s) 0~360°)	±5 m/s
Solar radiation collector		0~1800 (W/m^2^)	±5%
Air temperature collector		40~123.8 (°C)	±0.4°C 25°C
Air humidity collector		0~100% (RH)	±3.0% RH
Atmospheric pressure collector		0~1100 (hPa)	±0.3 hPa
Monitoring devices	Hach LDo	0~20 (mg/L)	±0.5%
Handheld device	Hq40d		±0.02°C

The four monitoring devices were regularly placed in the aquaculture pond one meter below the water surface, and the dissolved oxygen content and temperature data were collected every 10 minutes. The handheld instrument was used to collect the dissolved oxygen content data for sampling points 1 to 9 at three different depths (0.4 m, 1.0 m, and 1.6 m) in the water, but a depth of only 0.2 m was used to collect data for sampling points 10 to 19 (Fig 4B). The sampling of dissolved oxygen content using the handheld device was completed within 40 minutes. The data files included the sampling time, the three-dimensional coordinates of the sampling point in the aquaculture pond, and the dissolved oxygen content value. The automatic Meteorological Station was placed beside the aquaculture pond and used to sample the meteorological data. The file for the meteorological data included the sampling time, rainfall, wind speed, wind direction, solar radiation, air temperature, air humidity, and atmospheric pressure.

For this study, data samples were collected from June 23, 2015, to July 13, 2015. A total of 21 days of aquaculture dissolved oxygen content data were collected using the four monitoring devices and the one handheld instrument. To ensure the integrity of the experiment, dissolved oxygen content data were collected during different types of weather (sunny, cloudy, rain, etc.). Sixty-one samples were selected within one hour between 7:00 and 8:00 on July 6, 2015, to build the three-dimensional model of dissolved oxygen content in the aquaculture pond. Fifty-one samples were used to design and train the model; the remaining ten samples were used to compare the performance of the existing RBF interpolation model and the proposed SC-K-means-RBF interpolation model.

### Description of the overall three-dimensional prediction model

**Fig 5:**
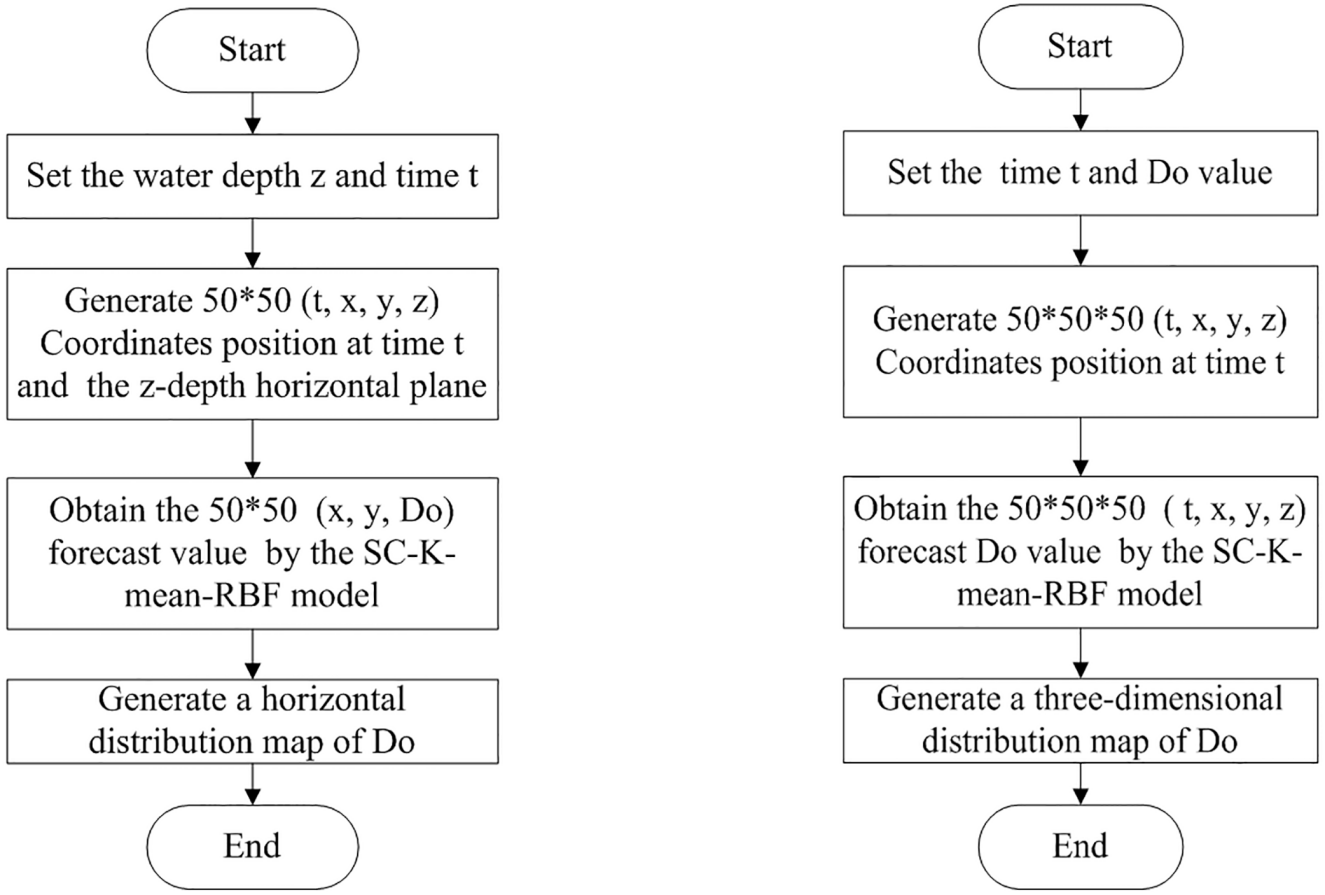
The process of the three-dimensional model for dissolved oxygen content.

In the RBF neural network, the input data X are a 4-dimensional vector, X = [*x*_1_, *x*_2_, *x*_3_, *x*_4_]^*T*^. For the RBF spatial interpolation of the dissolved oxygen content of the aquaculture pond, *x*_1_ denotes the sampling time, and *x*_2_, *x*_3_, *x*_4_ denote the three-dimensional coordinates of the sampling points in the aquaculture pond. The output data y denote the dissolved oxygen content. To generate the horizontal and vertical dissolved oxygen content distributions, the breeding ponds were evenly divided between the horizontal and vertical planes. The details of the horizontal level dissolved oxygen content distribution are as follows (Fig 5). First, the water depth z and time t were set, and then 50 * 50 (t, x, y, z) coordinates position were generated at time t and the z-depth. Second, 250 coordinates were selected, and the SC-K-means-RBF model was used to predict the dissolved oxygen content value. Finally, the MATLAB toolbox was used to complete the horizontal prediction display.

### Three-dimensional prediction of dissolved oxygen content

#### Interpolation accuracy evaluation

**Fig 6:**
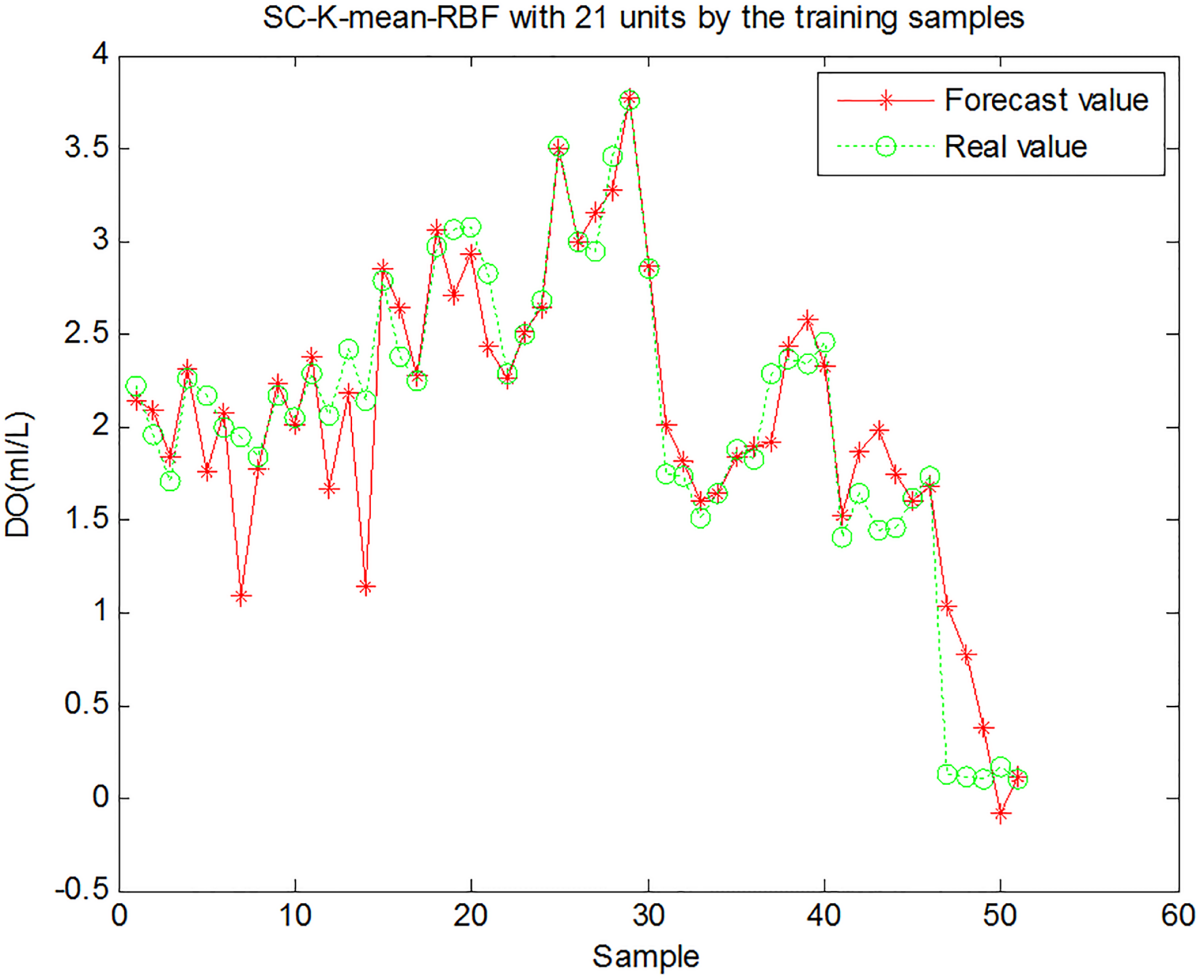
Training results for dissolved oxygen content prediction using the SC-K-means-RBF model.

To verify the interpolation performance of the proposed SC-K-means-RBF model, 61 datum points from the sampling data set taken between 7:00 and 8:00 on July 6 were selected to train and test the interpolation model. The first 51 data values were used for model training, and the remaining 10 data sets were used for model testing. The forecasting results showed that the proposed SC-K-means-RBF had good forecasting performance (Fig 6). The SC-K-means-RBF model had a very small root mean square error (RMSE) and MAE during the training and testing stage. In addition, the results showed consistently good correlation throughout training (>0.93) and testing (>0.81), as shown in Table 2.

**Table 2:** Error statistics of the four forecasting models.

Method	SC-K-means-RBF (training)	SC-K-means-RBF (testing)	Standard RBF					IDW	Kriging
Hidden units	21	21	10	15	20	21	22	-	-
RMSE	0.3072	0.4929	0.7022	0.6165	0.5646	0.6482	0.5344	0.6447	0.3800
MAE	0.2000	0.3484	0.4841	0.4152	0.3628	0.3932	0.3579	0.4442	0.3376
R	0.9309	0.8098	0.5568	0.6541	0.7311	0.6903	0.7525	0.6077	0.9175
D	0.9998	0.9879	0.9725	0.9711	0.9993	0.9521	0.9265	0.9993	0.9650

**Fig 7:**
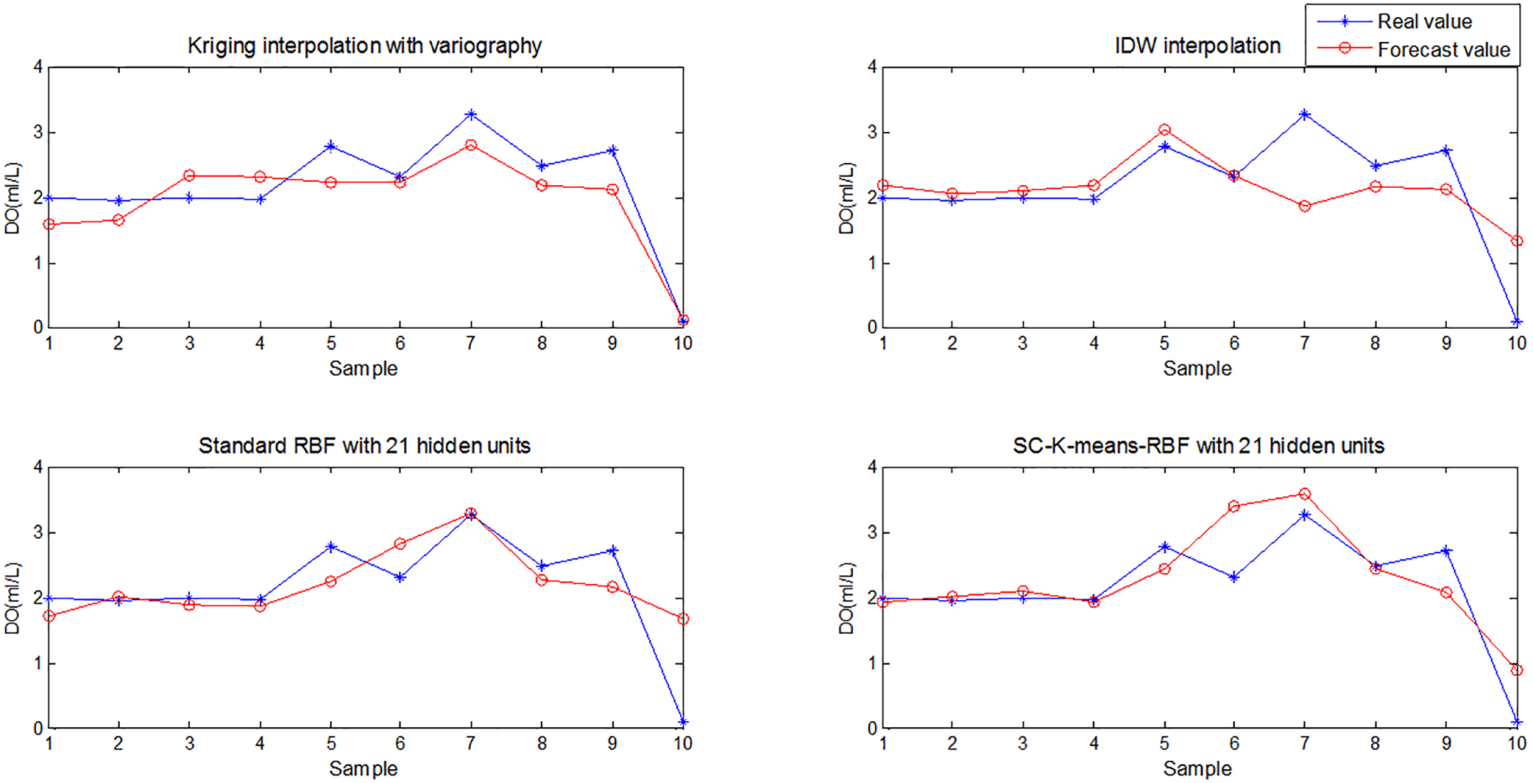
Comparison of the dissolved oxygen content forecasting values obtained by SC-K-means-RBF and other methods.

Comparing the three figures, the standard RBF interpolation method with ten hidden units had nearly the same interpolation effect as the proposed method. The importance of the number of hidden units in the RBF interpolation method is illustrated in Fig 7. Although the proper number of hidden units in the RBF interpolation can be obtained with trial-and-error, which has a smaller RMSE value and almost the same forecasting accuracy as the proposed interpolation method, it is not a practical interpolation method for 3D modeling of dissolved oxygen content in an aquaculture pond. The standard RBF interpolation method would be a waste of time and would not guarantee the accuracy of the 3D model due to the abundance of data. The validity, stability, and accuracy of the proposed 3D interpolation modeling method are thus clearly demonstrated. Therefore, the SC-K-means-RBF interpolation was chosen as the method for constructing a 3D model of dissolved oxygen content in the aquaculture pond.

To analyze the forecasting capacity of the hybrid model based on **SC-K-means-RBF**, the standard RBF, inverse distance weighted (IDW) and Kriging with variography methods were selected for comparison. The variogram is a basic tool for the Kriging method to describe the spatial autocorrelation structure of the explored variable and obtain the weights. The spherical function was selected as the Kriging variogram function in this study. The variogram analysis had a basic lag distance of 100 m and lag distance number of 20 units. To analyze and evaluate the prediction performance of the presented model for dissolved oxygen content, the testing samples were used for validation. The RMSE, mean absolute error (MAE), coefficient of correlation (R) and Willmott index of agreement (D) of the training and testing results were used to evaluate the forecasting capacity of the four models shown in Table 2. The performance indexes were calculated from the following equations:

$$\text{RMSE}\;=\;\sqrt{\frac{\sum_{1}^{N}{\left(X_{i}^{*}-X_{i}\right)}^{2}}{N}}$$ (12)

$$\text{MAE}\;=\;\frac{1}{N}\sum_{1}^{N}\left|X_{i}^{*}-X_{i}\right|$$ (13)

$$R\;=\;\frac{\sum_{1}^{N}\left(X_{i}-\bar{X})(X_{i}^{*}-\overset{-}{X^{*}}\right)}{\sqrt{\sum_{1}^{N}{\left(X_{i}-\bar{X}\right)}^{2}\sum_{1}^{N}{\left(X_{i}^{*}-\overset{-}{X^{*}}\right)}^{2}}}$$ (14)

$$D\;=\;1-\frac{\sum_{1}^{N}{\left(X_{i}-X_{i}^{*}\right)}^{2}}{\sum_{1}^{N}{\left(\left|X_{i}-\bar{X}|+|X_{i}^{*}-\bar{X}\right|\right)}^{2}}$$ (15)

where *X*_*i*_ is a real value, $X_{i}^{*}$ is the forecast value of the interpolation method, and *N* denotes the number of test data. The model with the smallest values of RMSE and MAE is the best. In addition, a value of the coefficient of correlation R to one indicates a better fit.

The RMSE and MAE of the proposed SC-K-means-RBF interpolation method were 0.4929 and 0.3484, respectively, and this method had the lowest RMSE and MAE values compared with the other three methods in Table 2. The proposed SC-K-means-RBF interpolation method chose 21 hidden nodes by combining the subtractive clustering and K-means clustering methods, and five values of hidden nodes (e.g., 10, 15, 20, 21, and 22) were selected to design the standard RBF interpolation model using trial and error. The coefficient of correlation (R) of the SC-K-means-RBF method was 0.8098, closest to 1. Thus, this prediction was the most accurate. All index values of IDW are larger than those of the presented method and the Kriging method. The Kriging with variogram function yielded good interpolation results but was inferior to the SC-K-means-RBF method. The values of RMSE and MAE of the standard RBF interpolation method were greater than those of the proposed method. The RMSE and MAE values of the standard RBF interpolation method were also larger than the proposed method when 21 hidden numbers were selected. The mean of the five RMSE values was 0.6132, and the proposed SC-K-means-RBF interpolation method yielded an average RMSE reduction of 19.62% compared to the standard RBF interpolation method. The RMSE and MAE values of IDW were 0.6447 and 0.4442, respectively, also larger than those of the proposed method. By contrast, the Willmott index of agreement (D) values of IDW and standard RBF with 21 hidden units were both 0.9993, better than that of the presented model. The Willmott index of agreement (D) value of the SC-K-means-RBF was 0.9879, illustrating the relatively good generalizability of the presented model.

#### Three-dimensional model of dissolved oxygen content

The proposed 3D spatial interpolation method was applied to establish the 3D model of the dissolved oxygen content in the aquaculture pond. The meteorological data from the corresponding time were selected to test the model’s accuracy and analyze the 3D model of the dissolved oxygen content of the aquaculture pond. The training and test data for the RBF network and the meteorological data for the corresponding time are shown in Tables 3 and 4.

**Table 3:** Training and test data.

Time	X (m)	Y (m)	Z (m)	Dissolved oxygen (mg/L)
7:07	15	25	0.4	2.22
7:07	15	25	1	1.95
7:07	15	25	1.6	1.7
7:10	22.5	25	0.4	2.26
…				

**Table 4:** Meteorological data.

Time	Rainfall (mm)	Wind speed (m/s)	Wind direction (°)	Solar radiation (w/*m*^2^)	Air temperature (°C)	Relative humidity (%)	Atmos. Pressure (KPa)
2015-07-06 07:00:04	0.0	2.19	262.06	77.85	19.92	88.54	100.96
2015-07-06 07:10:06	0.0	1.94	263.76	81.32	19.93	88.05	100.96
2015-07-06 07:20:08	0.0	1.9	260.12	95.67	20.01	88.44	100.96
2015-07-06 07:30:08	0.0	1.94	271.56	100.99	20.11	87.77	100.96
2015-07-06 07:40:06	0.0	1.23	270.87	89.23	20.14	87.08	100.96
2015-07-06 07:50:01	0.0	1.23	268.65	116.01	20.32	86.56	100.96
2015-07-06 08:00:02	0.0	1.61	271.59	134.87	20.38	83.64	100.96

The proposed SC-K-means-RBF network was trained using the sampling data in Table 3. Interpolation values of all points in the space of the aquaculture pond were generated using the trained model. Then, the 3D model of the dissolved oxygen content in the aquaculture pond was built. Additionally, the introduction of the time parameter revealed changes in the dissolved oxygen content in the aquaculture pond over time in the 3D model.

#### Horizontal forecast presentation

**Fig 8:**
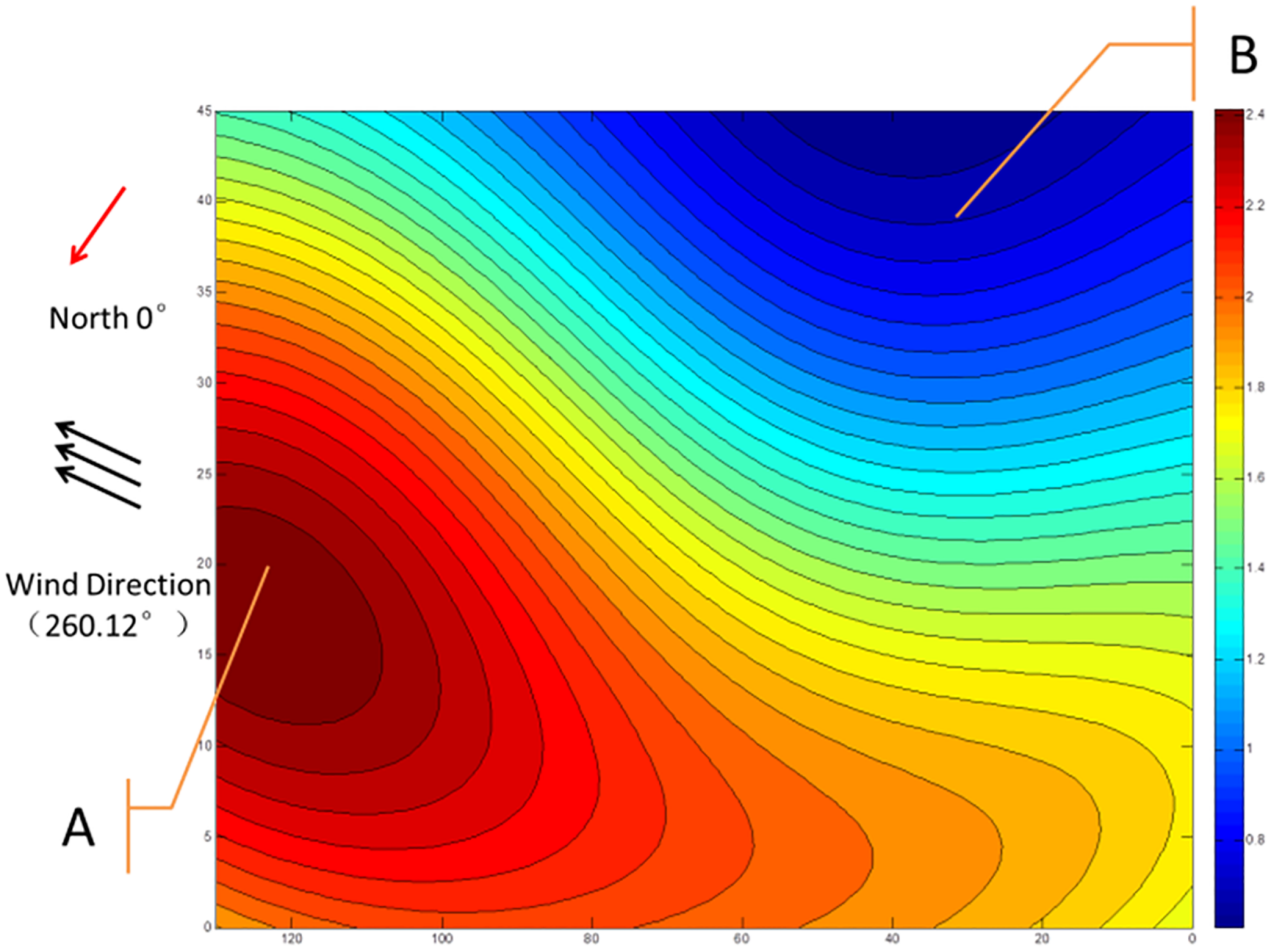
Cross-section of the spatial distribution of dissolved oxygen in the aquaculture pond.

In the aquaculture pond, the dissolved oxygen content distribution is uneven. At the same level, the distribution of dissolved oxygen is affected by wind speed, pond boundaries, and other factors. The data were taken from one meter below the surface of the water at 7:20 on July 6, 2015. The color change from yellow to blue denotes that the concentration of dissolved oxygen content changed from high to low. Dissolved oxygen was low at the uptake and high at the downcomer. Dissolved oxygen was relatively high at the leeward edge of the pond. Therefore, point B had a low level of dissolved oxygen content, and point A had a high level of dissolved oxygen content (Fig 8).

#### Three-dimensional prediction

**Fig 9:**
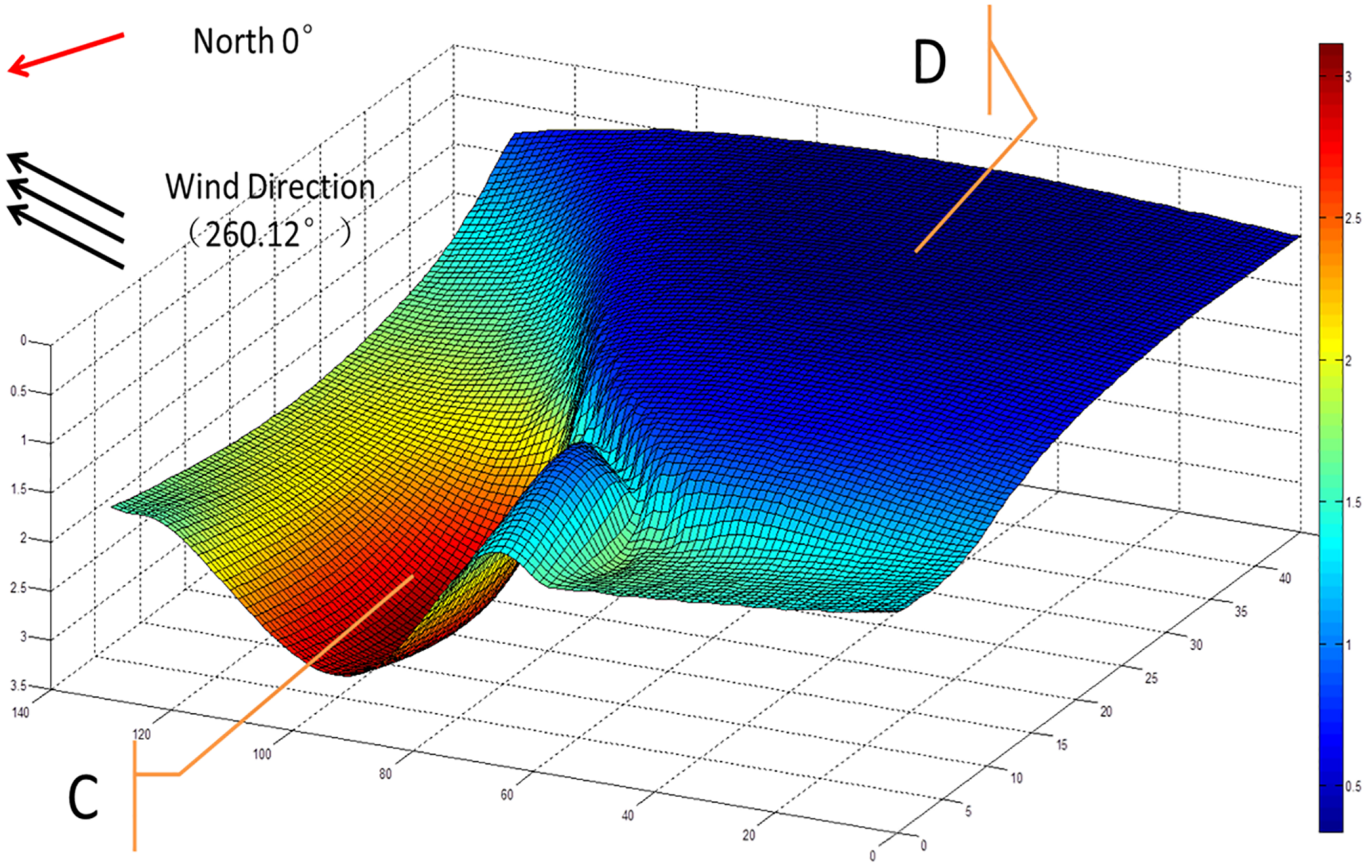
Curved surface of dissolved oxygen of 2 mg/L.

In the vertical direction, the color change from yellow to blue denotes that the depth of dissolved oxygen content of 2 mg/L changed from deep to shallow. At point C of the aquaculture pond, the dissolved oxygen content reached 2 mg/L at a low depth; however, at point D of the aquaculture pond, the dissolved oxygen content reached 2 mg/L at a relatively shallow depth, close to the water surface (Fig 9). These observations indicate that there was more dissolved oxygen content in the C direction of the aquaculture pond and less dissolved oxygen content in the D direction of the aquaculture pond. The dissolved oxygen content gradually decreased as the depth increased.

We analyzed the distribution of dissolved oxygen content in the aquaculture pond in connection with the meteorological data in Table 3. The wind direction was 260.12°, as shown in Table 4, indicating that the wind was blowing from the southwest to the northeast at 7:20 on July 6, 2015. The general direction of the wind is marked in Figs 8 and 9. The general direction from point B to point A in Fig 8 and point D to point C in Fig 9 is similar direction to the wind direction, as shown in the two figures. Therefore, it can be concluded that the 3D and 2D distributions of dissolved oxygen content in the aquaculture pond are correlated with the wind direction.

## Conclusion

The SC-K-means-RBF model is a three-dimensional spatial interpolation method based on RBF incorporating subtractive clustering and K-means for dissolved oxygen content prediction and display. The method can identify changing trends and provide guidance for aquaculture. The SC-K-means-RBF interpolation method, which has a clear principle and simple structure, provides a simple method for 3D modeling the spatial distribution of dissolved oxygen content in an aquaculture pond. Combining the subtractive clustering method and K-means clustering method increases the accuracy of obtaining the number of hidden units for the RBF neural network. The results illustrate the validity of the proposed SC-K-means-RBF interpolation method by comparing the RMSE and MAE values of the proposed method with the standard RBF interpolation method. For example, the standard RBF interpolation achieves RMSE values from 0.5344 to 0.7022 with different hidden units, and the proposed SC-K-means-RBF interpolation method achieves lower RMSE values. The comparison of the prediction results of different traditional models validated the effectiveness and accuracy of the proposed hybrid SC-K-means-RBF model for the three-dimensional prediction of dissolved oxygen content.

The proposed SC-K-means-RBF interpolation was then applied to analyze the distribution of dissolved oxygen content in the aquaculture pond in connection with meteorological data. The analysis showed that the 3D and 2D distributions of dissolved oxygen content in the aquaculture pond were correlated with wind direction. Therefore, the proposed SC-K-means-RBF interpolation method is an effective alternative to the existing method. The general direction of point B to point A and point D to point C is not identical to the wind direction, as indicated in Figs 8 and 9. This observation may be due to a lack of meteorological parameters in the input layer of the proposed RBF spatial interpolation method. In future studies, other essential parameters could be added to improve the accuracy of the model. Additionally, the three-dimensional distribution of dissolved oxygen can provide a reference for feeding strategies, as the oxygen solubility can affect feeding.

## Supporting information

S1 File The subtractive clustering (SC)-K-means-RBF model. (M)

S2 File The training and testing data. (XLSX)
